# Parkin overexpression modulates gut-microbiota composition during aging in *Drosophila melanogaster*

**DOI:** 10.3389/fmicb.2025.1672083

**Published:** 2025-09-30

**Authors:** Larisa Sarghie, Paula Istvan, Ricardo Aparicio, David W. Walker, Robi Tacutu, Marius Surleac

**Affiliations:** ^1^Institute of Biochemistry of the Romanian Academy, Bucharest, Romania; ^2^Department of Integrative Biology and Physiology, University of California, Los Angeles, Los Angeles, CA, United States; ^3^European Virus Bioinformatics Center (EVBC), Friedrich Schiller University of Jena, Jena, Germany; ^4^The Research Institute of the University of Bucharest, Bucharest, Romania; ^5^National Institute for Infectious Diseases "Matei Balș", Bucharest, Romania

**Keywords:** Parkin gene, lifespan, antimicrobial peptides (AMP), gut micobiome, *Drosophila melanogaster*

## Abstract

**Background:**

The gut microbiota plays a key role in host health during aging, influencing metabolism, immune function, and lifespan. In older individuals, the microbial community often becomes less diverse and more unstable, which can contribute to chronic inflammation and increased disease risk. Parkin, an E3 ubiquitin ligase, is known to extend lifespan when overexpressed in *Drosophila melanogaster*, but it's still unclear whether it also influences the gut microbiota during aging and whether this might contribute to its longevity effects.

**Methods:**

To investigate this, we used an inducible genetic system to overexpress Parkin in adult *D. melanogaster*. Midguts were collected at four time points: days 10, 30, 45, and 60, and bacterial DNA was extracted for 16S rRNA amplicon sequencing to characterize microbiota composition and diversity. To assess the functional impact of these microbial communities, homogenates from Parkin-overexpressing and control flies were fed to germ-free wild-type recipients, followed by monitoring of lifespan and expression of antimicrobial peptides.

**Results and discussion:**

Parkin overexpression resulted in age-dependent changes in gut microbiota composition and diversity. Community structure shifted significantly, with more pronounced differences observed in older flies. When fed to germ-free wild-type flies, homogenates from middle-aged and old control flies reduced the median lifespan. In contrast, the microbiome from Parkin-overexpressing flies was more similar to that of young flies. It did not reduce median lifespan and did not trigger the proinflammatory response seen with the control microbiome. Our findings suggest that Parkin promotes a gut microbial environment that is more balanced and less inflammatory, which may support healthier aging.

**Conclusion:**

This study demonstrates that Parkin overexpression influences gut microbiota composition in a way that may be beneficial to host health during aging. The microbial communities associated with Parkin-overexpressing flies were not only distinct but also functionally advantageous, reducing immune activation and extending median lifespan in germ-free recipients. To our knowledge, this is the first study to use Parkin overexpression to explore potential Parkin-related changes in the gut microbial community, changes that were captured dynamically at four different stages of the *D. melanogaster* lifespan.

## 1 Introduction

Over the past decade, accumulating evidence has demonstrated that the gut microbiota plays a central role in regulating host health and longevity. It influences a broad spectrum of physiological processes, including metabolic function, immune regulation, and neurological activity, with effects observable from early development to old age (Donati Zeppa et al., 2022; Bosco and Noti, 2021). As we grow older, the balance and stability of this microbial ecosystem begin to shift. These age-related changes often lead to a loss in microbial diversity and an increase in instability, which have been linked to weakened gut barrier function, chronic low-grade inflammation, and a higher risk of developing age-associated diseases (Donati Zeppa et al., 2022; Clark et al., 2015). Its rapid life cycle, low-complexity gut microbiota, and striking genetic overlap with humans, about 75% of disease-related genes have fly orthologues, combined with the ease of creating targeted mutants, transgenic lines and germ-free/gnotobiotic cohorts, make *Drosophila melanogaster* a premier model for dissecting host–microbe interactions (Matthews et al., 2020; Lee et al., 2019; Douglas, 2018). Notably, generating axenic flies, when achieved without negatively affecting host development, has been shown to significantly extend lifespan (Lee et al., 2019).

Internal cellular maintenance systems play a vital role in preserving tissue function over time via autophagy, a cellular mechanism that allows for the removal and recycling of damaged or unnecessary components (Aman et al., 2021). A particularly important form of autophagy is mitophagy, the selective degradation of dysfunctional mitochondria. This process ensures mitochondrial quality control, limiting the accumulation of reactive oxygen species and maintaining energy balance. The most well-characterized mitophagy pathway involves the proteins PINK1 and Parkin (PRKN gene) (Aman et al., 2021; Harrington et al., 2023). Research suggests that Parkin is involved in mitochondrial quality control by interacting with the fission and fusion machinery to promote the removal of damaged mitochondria (Rana et al., 2013; Ashrafi and Schwarz, 2013; Youle and van der Bliek, 2012). Recent work shows that the gut microbiome can modulate the Pink1/Parkin circuit in colonocytes through the secretion of microbial short-chain fatty acids (SCFAs), bile-acid derivatives, and indole metabolites (Gou et al., 2025). Under such conditions, the epithelium enjoys high ATP output, low reactive-oxygen-species (ROS) load and robust IgA secretion, features that, in turn, favour a eubiotic, healthy microbiota. Dysfunctions of mitochondrial quality control caused by genetic defects in Pink1, chronic nutrient excess, endocrine shifts, or environmental toxins have been also associated with local hypoxia, which increases the facultative anaerobes, driving dysbiosis (Pral et al., 2021). A vicious cycle ensues: pathobionts release lipopolysaccharides, p-cresol, and trimethylamine-N-oxide, all of which further impair mitochondrial respiration. Concomitantly, gut-derived hydrogen sulfide blocks cytochrome-c oxidase, tipping the balance toward excessive fission and inflammasome activation. The outcome is a feed-forward loop of epithelial injury, chronic low-grade inflammation, and progressive microbial imbalance, a pattern observed in inflammatory bowel disease, metabolic syndrome, and aging (Alula et al., 2023).

**Figure 1:**
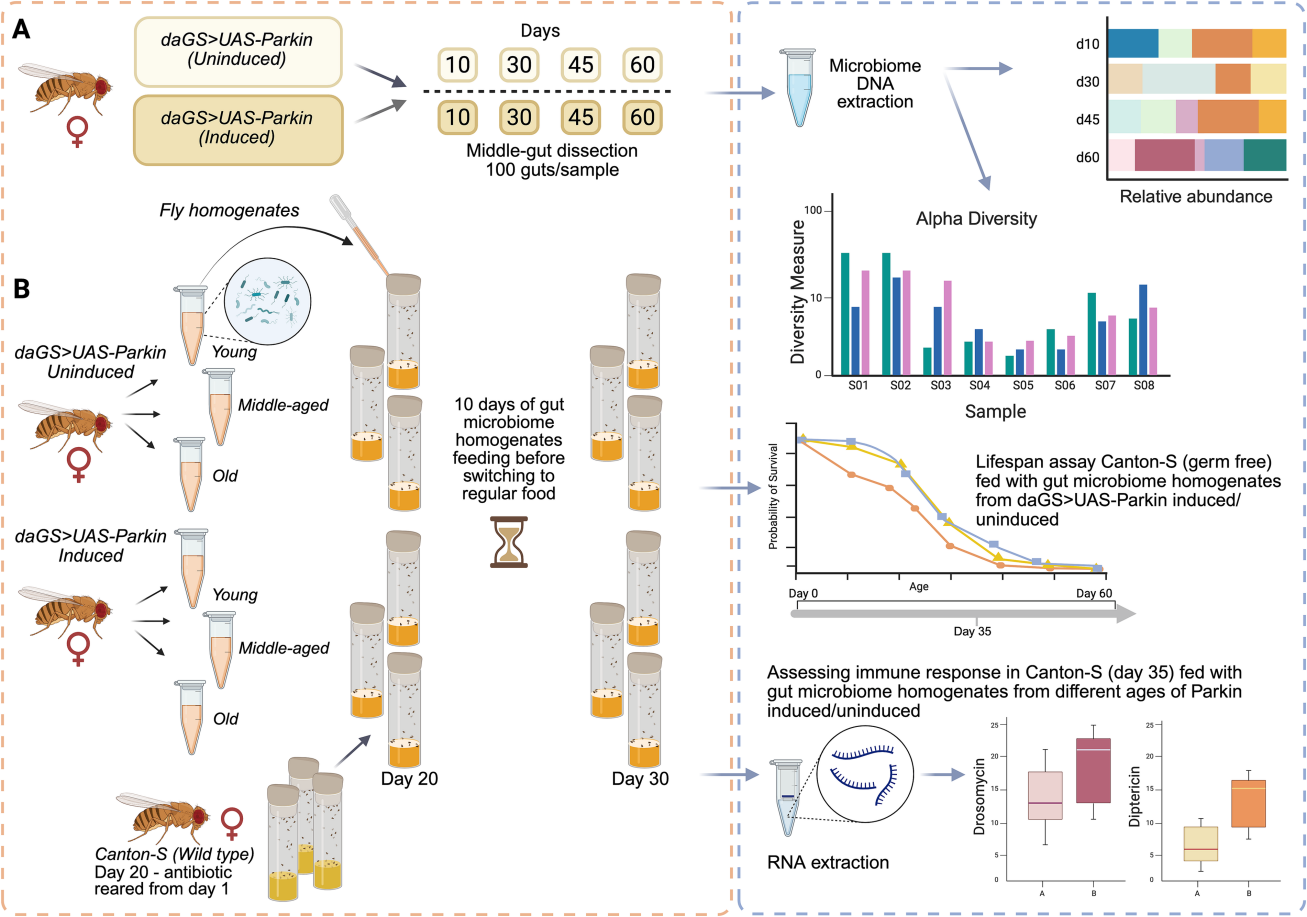
Experimental workflow diagram (created in https://BioRender.com): **(A)** A total of 100 midguts were dissected from *daGS>UAS-Parkin* female flies under two conditions: Uninduced (Control) and Parkin induced (OE), at four different time points (day 10, 30, 45, and 60). Microbial DNA was extracted, followed by 16S rRNA taxonomic profiling and diversity analysis. **(B)** To assess whether the microbiome from Parkin uninduced and induced flies influenced the host over time, homogenates were prepared from young (day 10), middle-aged (day 30), and old (day 60) flies. These homogenates were administered to axenic wild-type flies (Canton-S). After 10 days of feeding on the homogenates, Canton-S flies were transferred to regular food. Three parameters were analyzed after 10 days of homogenate feeding: Antimicrobial Peptide expression and 16S microbial load at day 35, and lifespan.

However, the interactions of mitochondrial quality control and aging with the gut microbiome remain poorly understood. *Drosophila melanogaster*, whose gut harbors only a few dominant taxa and whose Pink1/Parkin mitophagy pathway is highly conserved, combines gnotobiotic tractability with rapid, transgene-friendly genetics making it an ideal system for host–microbe–mitochondria studies (Broderick and Lemaitre, 2012). Upregulation of Parkin in adult *D. melanogaster* has been shown to increase both mean and maximum lifespan, while maintaining normal levels of reproductive output, physical activity, and food consumption (Rana et al., 2013). Flies with Parkin overexpression (Parkin OE) demonstrate low levels of K48-linked polyubiquitin chains and reduced protein aggregation as they age. Parkin OE also leads to decreased Mitofusin levels in aging flies, resulting in altered mitochondrial structure and increased mitochondrial activity (Rana et al., 2013; Si et al., 2019). In this study, we designed an experimental framework (Figure 1) to investigate how age-related changes in microbiome composition affect *D. melanogaster* lifespan and immune responses, and whether Parkin overexpression can modulate this relationship. Our results show that flies overexpressing Parkin exhibit a distinct microbial profile compared to control flies. Additionally, microbiota from aged control flies had a detrimental effect on lifespan when transferred to germ-free recipients at 20 days of age, whereas microbiota from Parkin-OE flies maintained a consistent, comparatively beneficial effect regardless of donor age. The microbiome from aged Parkin uninduced flies was associated with the activation of innate immune responses, a pattern that was not observed in flies with induced Parkin expression. These findings suggest that Parkin OE may contribute to healthier aging not only through mitochondrial quality control but also by shaping a eubiotic microbiota.

## 2 Materials and methods

### 2.1 Drosophila husbandry

The following lines were used Canton-S (BDSC 64349), *w^1118^; daughterless-GeneSwitch (daGS)* (obtained from H. Tricoire at Université Paris Diderot), and *UAS-Parkin* (provided by L. Pallanck, University of Washington, Seattle, WA). Flies were reared in vials containing cornmeal medium (9.1% cornmeal, 3.8% dextrose, 3% yeast, 1.9% sucrose, 1% agar, 1.1% acid mix (41.8% Propionic acid plus 4.15% Phosphoric acid in vol/vol), and 1.5% methylparaben (10% methylparaben in ethanol), all concentrations given in wt/vol). Flies were sorted under mild anesthesia induced by nitrogen. The population was synchronized and only females were collected on the third day after eclosion, allowing time for mating and sharing the microbiome.

### 2.2 Lifespan assay

Flies were maintained at a population density of 27–32 individuals per container and transferred to new vials every 2–3 days, during which mortality was recorded throughout their adult lifespan. To activate the gene switch (overexpressing the PRKN gene), RU486 (Cayman Chemical Company, cat#10006317) was initially dissolved in ethanol and then integrated into the food medium during preparation. The final concentration of RU486 administered was 5 μg/mL, while the control group’s food contained only ethanol (Clark et al., 2015; Rana et al., 2013).

### 2.3 Generation of axenic and re-association by adult feeding

Germ-free (axenic) *D. melanogaster* were generated by rearing Canton-S adults on food supplemented with a broad-spectrum antibiotic cocktail containing 500 μg/mL ampicillin, 50 μg/mL tetracycline, and 200 μg/mL rifamycin for 20 days, starting from the first day after eclosion (Brummel et al., 2004). During this period, flies were transferred to fresh vials containing antibiotics every 2–3 days, and the effectiveness of bacterial clearance was monitored by plating 50 μL of fly gut homogenates and vial suspensions on MRS agar plates, with successful germ-free status confirmed by the absence of microbial growth. After 20 days on antibiotics, female flies were transferred to regular food supplemented with gut microbiome homogenate from either *daGS>UAS-Parkin* control or Parkin-overexpressing donor flies collected at days 10, 30, or 60. Flies were maintained on this microbiota-supplemented food for 10 days, with the food replaced every 2–3 days to ensure microbial viability and consistency. Following this re-association period, flies were transferred to standard food and maintained under normal conditions until the end of the lifespan assay, with survival monitored until the death of the last individual.

### 2.4 Preparation of fly homogenate for re-association

Conventionally reared adult flies were surface sterilized by 75% ethanol as previously described (Ren et al., 2007) before homogenization to ensure only internal microbes were present in the homogenate. Surface-sterile flies were homogenized in sterile PBS using a motorized pestle (50 flies per 200 μL in a 1.5 mL tube). The resulting homogenates were pooled, and sterile PBS was added to adjust the final volume so that the microbial content of one fly was contained in 50 μL of PBS (i.e., 50 flies were diluted to a total volume of 2.5 mL). To preserve the homogenate for future use, 1/5 volume of sterile 80% glycerol was added, and the aliquots were stored at −80 °C. For adult feeding experiments, freshly prepared homogenate (equivalent to one fly in 50 μL PBS) was added to standard food vials and left to dry before use (Clark et al., 2015).

### 2.5 Gut microbiome extraction, library preparation, and sequencing protocols

For intestinal sample collection, 150 flies were surface-sterilized in small batches of 10 flies per 2 mL Eppendorf tube containing 70% ethanol. This was done to ensure sterility and to compensate for potential losses during dissection, allowing us to collect 100 intact intestines. Dissections were performed over ice in sterile PBS using sterile equipment. The dissection surface was swabbed with 75% ethanol between each sample. Intestinal dissections included the whole midgut, from the point of cardia to the Malpighian tubules. Care was taken to keep the full length of the gut intact to prevent loss of lumen contents as previously described (Clark et al., 2015).

Bacterial DNA was extracted from 100 dissected midguts from *daGS>UAS-Parkin* female flies under two conditions: uninduced (Control) and induced (OE), at different time points (day 10, 30, 45, and 60) using QIAamp® DNA Microbiome Kit. Prior to starting the kit protocol, the midguts were homogenized with a motor pestle in a 2 mL tube with 1,000 μL of sterile PBS and then proceeded with the standard QIAamp® DNA Microbiome Kit protocol.

Genomic DNA was quantified using the Qubit 4 fluorometer with the Qubit™ dsDNA HS Assay Kit (ThermoFisher Scientific). A total of 5 ng DNA per sample was used for PCR amplification of the V4 region of the 16S rRNA gene using primers 515F, E806R, and A806R. Amplicons were sequenced on the Illumina MiSeq platform (2 × 250 bp paired-end reads). Raw reads were processed using the CosmosID-HUB 16S pipeline, which includes quality trimming, merging of paired-end reads, and conversion to FASTA format.

### 2.6 qPCR protocols

DNA samples for qPCR of the 16S ribosomal RNA gene were prepared as described above. RNA extractions for antimicrobial peptide gene expression analysis were performed using TRIzol (Invitrogen), following the manufacturer’s instructions. Each sample consisted of five flies, and a total of five samples were used. Flies were surface sterilized with 75% ethanol as previously described by Ren et al. (2007), and then homogenized in sterile PBS using a motorized pestle. cDNA synthesis was carried out using the First Strand cDNA Synthesis Kit from Fermentas. PCR was performed with Power SYBR Green master mix (Applied Biosystems) on an Applied Biosystems 7300 Real Time PCR system. Cycling conditions were as follows: 95 °C for 10 min; 95 °C for 15 s then 60 °C for 60s, cycled 40 times. All calculated gene expression values were normalized to the value of the loading control gene, Actin 5C. The primer sequences used to assess gene expression in this study were as follows: Act5C_F – TTGTCTGGGCAAGAGGATCAG, Act5C_R - ACCACTCGCACTTGCACTTTC; Dro_F – CCATCGAGGATCACCTGACT, Dro_R – CTTTAGGCGGGCAGAATG; Drs_F – GTACTTGTTCGCCCTCTTCG, Drs_R – CTTGCACACACGACGACAG; Dpt_F – ACCGCAGTACCCACTCAATC, Dpt_R – CCCAAGTGCTGTCCATATCC.

Universal primers for the 16S ribosomal RNA gene were used against variable regions 1 (V1F) and 2 (V2R), as previously published (Claesson et al., 2010). Taxon specific 16S primers were as follows: Bacilli_F – CGACCTGAGAGGGTAATCGGC, Bacilli_R – GTAGTTAGCCGTGGCTTTCTGG; Aceto_F - GCGAAGGCGGCAACCTGGCTC, Aceto_R - GCTTAACGCGTTAACTGCGAC; Gamma_F - GGTAGCTAATACCGCATAACG, Gamma_R – TCTCAGTTCCAGTGTGGCTGG.

### 2.7 Bioinformatics and statistical analysis

Raw 16S rRNA sequencing data were processed in R using the *DADA2* (Callahan et al., 2016) pipeline to generate 16S sequence variants (ASVs). A *phyloseq* (v1.3.8) (McMurdie and Holmes, 2013) object was created, integrating metadata, OTU counts, and taxonomy data for 16S. Operational taxonomic units (OTUs) were identified using closed-reference picking at 97% sequence similarity against the CosmosID curated 16S database via QIIME. Final outputs included taxonomic classifications, OTU frequencies, and relative abundances, with optional comparative analysis through the CosmosID platform. Taxonomic profiles were also assigned using Metaxa2 (Bengtsson-Palme et al., 2015). Alpha diversity metrics, including species richness, Shannon diversity, and inverse Simpson indices, were computed using *vegan* and visualized with *ggplot2* (Wickham, 2016). The 20 most abundant taxa in 16S datasets were identified, with remaining taxa grouped as “Others.” For Beta diversity analysis, the *phyloseq* object was collapsed by Genus, and PCoA was performed on Bray-Curtis distances with Hellinger transformation using *MicrobiotaProcess* (v1.6.6) (Xu et al., 2023). PCoA plots and additional distance-based visualizations were produced with *MicrobiotaProcess*. All statistical analyses and data visualizations were carried out using GraphPad Prism version 10.4.0(527) GraphPad Software, La Jolla, CA, USA (GraphPad Software, 2023). Statistical significance was assessed using *p*-values, with the specific tests noted in the corresponding figure legends. For comparisons between two groups, unpaired t-tests were used when the data met the assumptions for parametric testing, while the Mann–Whitney U test was applied for non-parametric data. In cases involving more than two groups, one-way ANOVA followed by Tukey’s multiple comparisons test was used if parametric conditions were met. The number of biological replicates (n) for each experiment is indicated in the figures and their legends. Survival analyses were conducted using the log-rank (Mantel–Cox) test.

## 3 Results

### 3.1 Changes in gut microbiota composition with Parkin overexpression

The first hypothesis tested in this study was that Parkin OE can influence gut microbiome composition. To assess this, both taxon-specific quantitative PCR and 16S rRNA analysis were performed, comparing microbial profiles between control and Parkin-OE flies across multiple age points (Supplementary Figure 1). The results revealed significant age-dependent differences in microbial abundance, indicating that Parkin expression influences the composition and metabolic functions of the gut microbiota over time. To ensure that the observed shifts were due to Parkin OE rather than the mifepristone (RU486) inducer (used to activate the system), a control experiment was conducted using the *w1118; daGS* transgenic flies. These flies, which carry the same inducible driver but not the Parkin transgene, were fed either regular food or food supplemented with RU486. No significant differences in the microbiome composition were observed between the two conditions (Supplementary Figure 2), confirming that the microbial shifts in the experimental group were specifically driven by Parkin OE rather than the inducing agent itself.

### 3.2 Dynamics of microbial community composition

**Figure 2:**
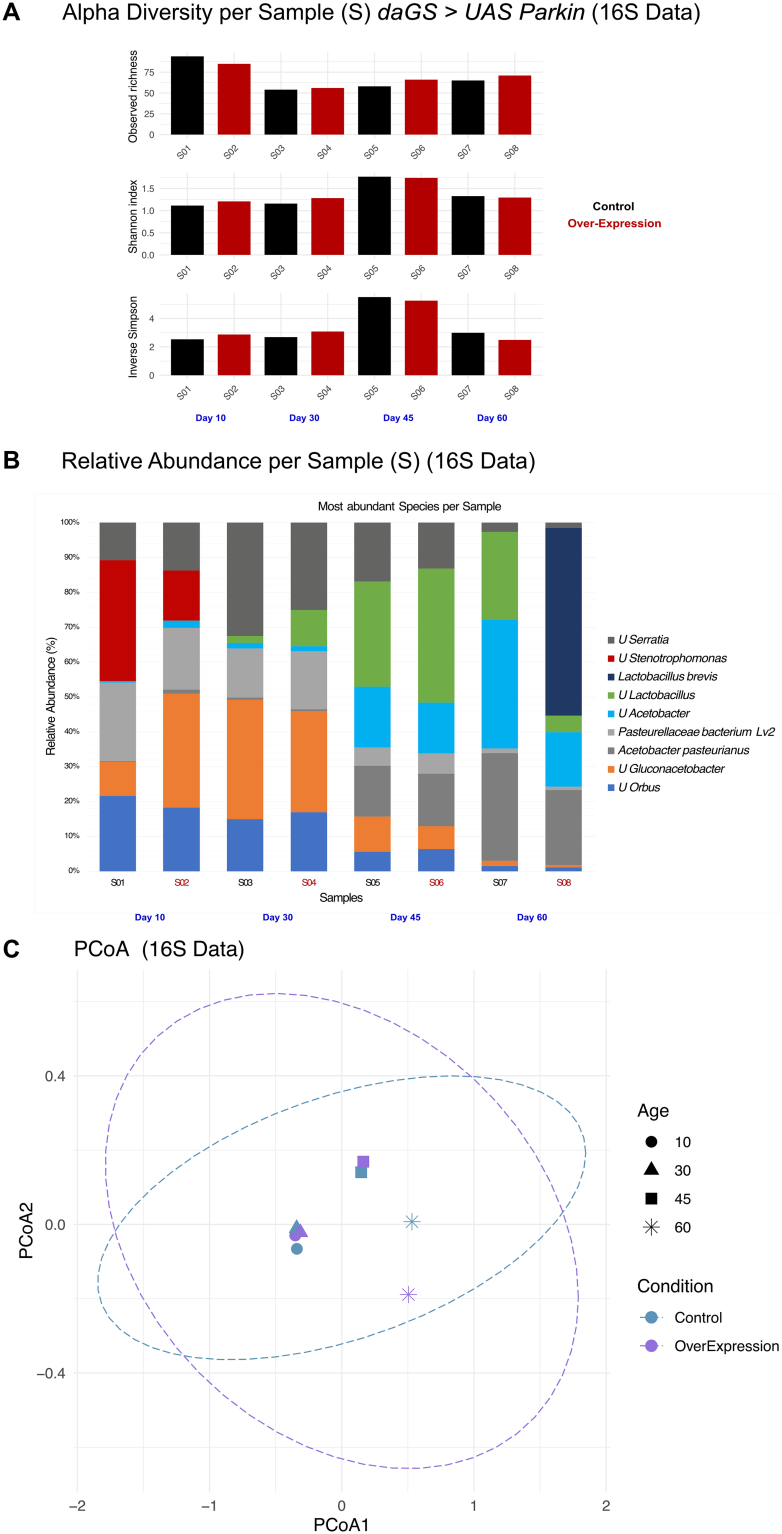
Alpha diversity metrics, relative abundance (%) and beta diversity based on 16S rRNA gene sequences across samples: **(A)** Alpha diversity metrics, including Observed richness, Shannon diversity, and Inverse Simpson diversity, for 16S datasets. **(B)** Relative abundance (%) of the top nine bacterial taxa identified in 16S rRNA gene sequencing data. Taxa are color-coded according to the legend, highlighting method-specific differences in taxonomic representation. **(C)** Beta diversity analysis based on Bray–Curtis dissimilarity, visualized via PCoA, comparing microbial community composition across samples. PCoA plot for 16S rRNA gene sequencing data, with samples grouped by *daGS>UAS Parkin* Control and *daGS>UAS Parkin induced* conditions. PERMANOVA, showing a significant effect of Age (𝑝 = 0.032).

Alpha diversity metrics revealed a sharp decline in microbial richness and evenness from early (S01–S02) to mid-adulthood (S03–S06), with partial recovery in late-stage flies (S07–S08). Notably, Parkin-overexpressing flies at day 60 (S08) maintained higher richness overall, while the diversity (based on Shannon and Inverse Simpson indices) is slightly decreased, compared to age-matched controls (S07). A similar effect is observed on younger flies (e.g., day 45). These changes would suggest that Parkin could alter the abundance of the dominant species in fly microbiota as reflected strongly by Inverse Simpson, and these changes are even more noticeable with age here, compared with the Shannon indices, where the effect is not as pronounced (Figure 2A)., In this context, evenness refers to how evenly individual reads are distributed across the different taxa present in a sample, where higher values indicate a more balanced community composition.

To explore overall differences in gut microbiota between groups, we performed a beta diversity analysis using Bray–Curtis dissimilarity and visualized the results with Principal Coordinate Analysis (PCoA) (Figure 2C). The plot shows that samples tend to group based on age in younger flies and based on Parkin expression on older flies. As can be noticed there is no significant difference between Control and Parkin overexpression in younger flies (e.g., days 10 and 30), but with age they start to behave differently, even showing a significant difference at day 60 between control and Parkin-overexpressing flies, in which they tend to separate. This suggests that aging strongly influences microbiota composition, and that Parkin overexpression may also contribute to shaping these changes. A statistical test (PERMANOVA) confirmed that age had a significant effect on microbial community structure (*p* = 0.032), supporting the idea that gut microbiota changes progressively with age and may be modulated by Parkin.

To better understand how the microbiota changed with age and Parkin expression, we looked at the relative abundance of the most common bacterial taxa in each sample using 16S sequencing data (Figure 2B, Supplementary Table 1). One of the most striking trends involved *Acetobacter pasteurianus*, a well-known gut symbiont in *Drosophila*. This species was already present in young flies, but its levels increased further with age and the effect was even more noticeable when paired with Parkin overexpression. It reached the highest abundance in the oldest flies, particularly in S08 (day 60, Parkin induced), where it accounted for more than half of the microbial community. These findings suggest that *A. pasteurianus* may thrive in the aging gut and that its expansion could be further promoted by Parkin overexpression. In contrast, *Gluconacetobacter* species were more abundant in the younger groups and gradually declined in older flies, and this effect was even more pronounced with Parkin overexpression. Their higher presence in S01 (day 10, Parkin uninduced) to S04 (day 30, Parkin induced) suggests they may play a more active role in early or middle stages of life but are eventually replaced or outcompeted in aged flies. The genus *Pasteurella*, including *Pasteurellaceae bacterium Lv2*, followed a similar age-related pattern. It was prominent in the microbiota of young and middle-aged flies but declined with age. A particularly interesting finding emerged in S08 (day 60, Parkin induced), where *Lactobacillus brevis* was strongly enriched. While barely undetectable in other groups, *L. brevis* made up slightly over 5 % of the total community in aged Parkin-overexpressing flies at day 60. This suggests that Parkin may play a role in shaping a gut environment that favors the expansion of this specific species later in life. Other taxa (e.g., *unclassified Acetobacter, unclassified Lactobacillus*) increased their levels with age while others decreased it (e.g., *unclassified Gluconacetobacter*, *unclassified Orbus*). These fluctuations could reflect individual variation, changes in gut physiology, or shifts in microbe–microbe interactions that occur with age or Parkin activity.

### 3.3 Microbiome reassociation and lifespan assay

**Figure 3:**
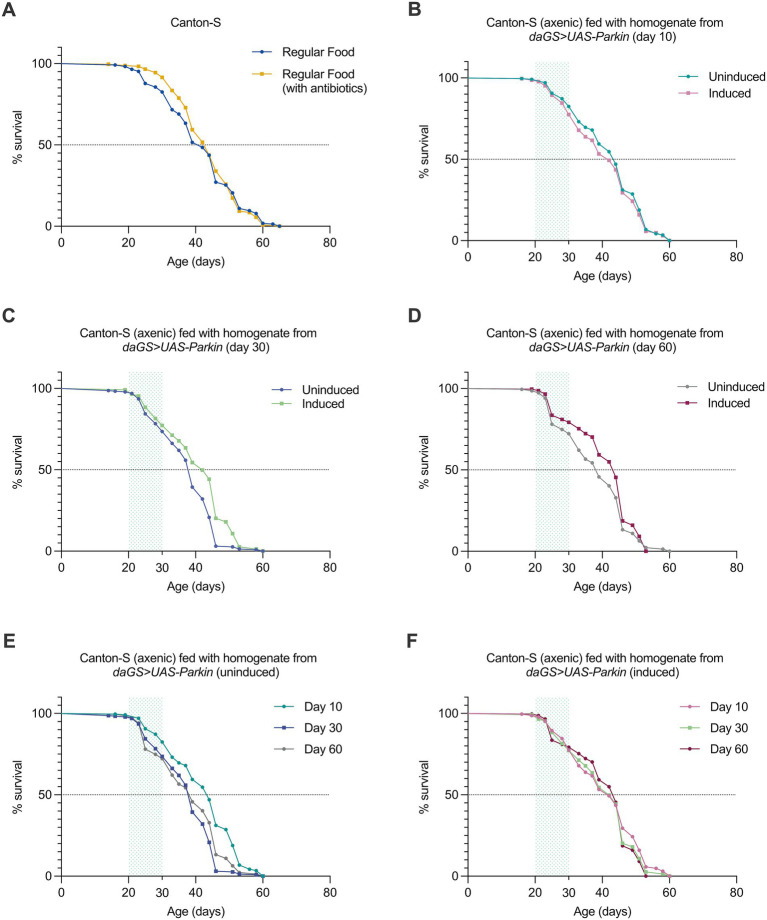
Homogenates from *daGS>UAS-Parkin* overexpression flies prevents lifespan decline when fed to germ-free Canton-S flies. **(A)** Survival curves of Canton-S female control flies reared on standard food (*n* = 229) or antibiotics (*n* = 236) throughout their lifespan (*p* = ns). **(B)** Survival curves of Canton-S female flies fed with young gut microbiome homogenate (day 10) from *daGS>UAS-Parkin* uninduced (*n* = 234) and induced (*n* = 227) donors (*p* = ns). **(C)** Survival curves of Canton-S female flies fed with middle-aged gut microbiome homogenate (day 30) from uninduced (*n* = 231) and induced (*n* = 233) *daGS>UAS-Parkin* donors (*p* < 0.0001). **(D)** Survival curves of Canton-S female flies fed with old gut microbiome homogenate (day 60) from uninduced (*n* = 219) and induced (*n* = 231) *daGS>UAS-Parkin* donors (*p* = 0.012). **(E)** Survival comparison of Canton-S female flies fed with gut microbiome homogenates from uninduced donors at young (*n* = 234), middle-aged (*n* = 231), and old (*n* = 219) time points (*p* < 0.0001). **(F)** Survival comparison of Canton-S female flies fed with gut microbiome homogenate from *daGS>UAS-Parkin* induced donors at young (*n* = 227), middle-aged (*n* = 233), and old (*n* = 231) time points (*p* = ns). All *p*-values were calculated using the log-rank (Mantel–Cox) test. The shaded area indicates the period during which flies were fed with the donor microbiome.

To investigate how the microbiomes of Parkin induced and uninduced flies influence host lifespan, we performed a microbiome reassociation experiment using germ-free Canton-S flies. The control strain Canton-S was depleted of its microbiome by being raised for 20 days on food supplemented with antibiotics (500 μg/mL ampicillin, 50 μg/mL tetracycline, and 200 μg/mL rifamycin) (Brummel et al., 2004). On day 20, flies from each vial were randomly tested to confirm the absence of gut bacteria. To ensure that antibiotics did not influence lifespan, we compared the lifespan of Canton-S on antibiotic media with that on regular food, observing no significant difference (Figure 3A).

To test whether the gut microbiome itself, shaped by age and Parkin expression, could influence lifespan independently of any direct genetic manipulation, we transferred microbiota from *daGS>UAS Parkin* donor flies with or without Parkin induction at different ages into 20-day-old germ-free wild type flies. By using unmodified hosts, we aimed to isolate the effects of the microbial community alone. After 10 days of microbiota exposure, we monitored the flies’ lifespan to assess the impact of these different microbial environments. Flies fed with homogenate from young (day 10) control and Parkin OE flies exhibited similar survival curves, with no difference in overall survival (Figure 3B). However, flies that received the microbiome from middle-aged (day 30) control flies showed a significantly reduced lifespan (*p* = 0.0001) compared to those receiving from Parkin OE flies. This suggests that the microbiome from 30-day-old control flies may negatively impact host longevity (Figure 3C). Similarly, flies fed with microbiome from old (day 60) control flies also showed a significantly shorter overall survival (*p* = 0.012) compared to those receiving gut microbiome homogenates from old Parkin OE flies (Figure 3D).

Overall, the gut microbiome homogenates from middle-aged and old control flies reduced the median lifespan of Canton-S flies, while those from Parkin OE flies did not show the same negative effect. Lifespan curves for Canton-S flies fed gut microbiome homogenates from control flies at days 10, 30, and 60 revealed a clear shift toward earlier mortality with age, despite similar maximum lifespans across groups. The gut microbiome homogenates from both day 30 and day 60 led to a significant reduction in survival compared to day 10 (*p* < 0.0001) (Figure 3E). In contrast, Canton-S flies receiving gut microbiome homogenates from Parkin OE donors at the same time points showed no such decline, indicating that the aged microbiome from Parkin OE flies does not negatively impact wild-type flies lifespan (Figure 3F).

### 3.4 The impact of microbiome transfer on immune response

**Figure 4:**
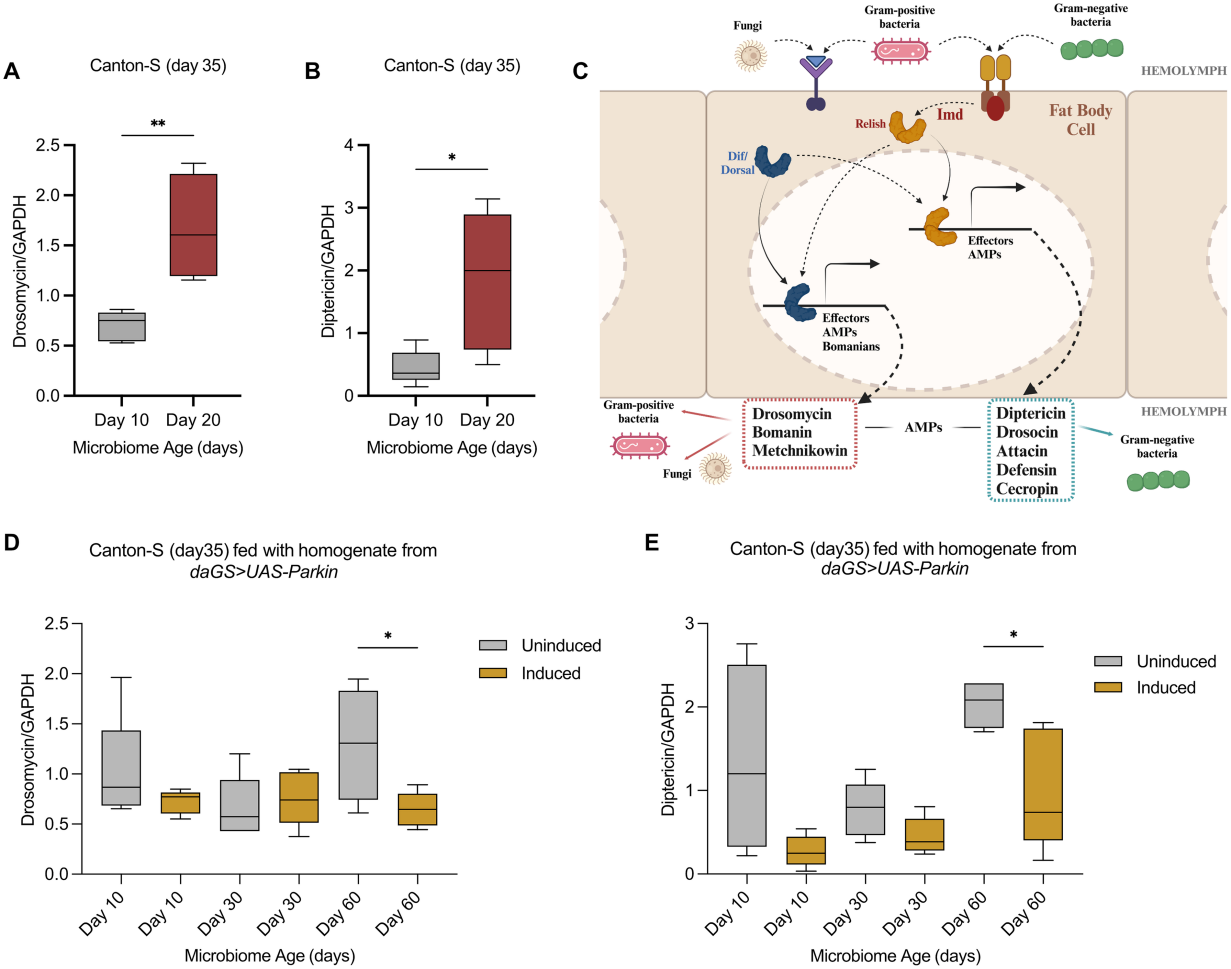
Microbiome Transfer Influences Antimicrobial Peptide Expression in Aged Drosophila **(A)** Expression levels of Drosomycin in 35-day-old Canton-S female flies following gut microbiome homogenates transfer from young and middle-aged Canton-S donors (**p = 0.0057). **(B)** Expression levels of Diptericin in 35-day-old Canton-S female flies after receiving gut microbiome homogenates from young and middle-aged Canton-S donors (*p = 0.0252). **(C)** Schematic representation of Drosophila immune response (adapted from Hanson and Lemaitre, 2020; created with BioRender: https://BioRender.com/q02f682). **(D)** Drosomycin levels in 35-day-old Canton-S female flies that received gut microbiome homogenates from *daGS>UAS-Parkin* donors. (*p = 0.0428). **(E)** Diptericin expression in 35-day-old Canton-S flies following gut microbiome homogenate transfer from *daGS>UAS-Parkin* donors. (*p = 0.0302). Panels **A**, **B**, **D**, and **E** represent data from five biological replicates per condition, with each replicate comprising five biologically independent Canton-S female flies.

One key mechanism by which the gut microbiome may influence host lifespan is through activation of the immune response, which regulates the production of antimicrobial peptides (AMPs) in *Drosophila*. AMPs such as Drosomycin and Diptericin are critical effectors of the innate immune response and are expressed in response to bacterial signals (Hanson and Lemaitre, 2020). While the Imd pathway is primarily triggered by Gram-negative bacteria and regulates Diptericin expression, the Toll pathway responds mainly to Gram-positive bacteria and fungi, mediating the expression of Drosomycin (Valanne et al., 2011; Hanson and Lemaitre, 2020). The AMP production process involves pattern recognition receptors like PGRP-LC and PGRP-LE, which detect bacterial peptidoglycans and trigger downstream signalings, ultimately activating the NF-κB transcription factors Relish and DIF (Figure 4C). These pathways become increasingly active with age, often leading to chronic low-grade inflammation and tissue dysfunction (Hanson and Lemaitre, 2020). To determine whether age-related changes in the microbiome affect the host immune response, we measured the expression of two antimicrobial peptides, Drosomycin and Diptericin. To assess the impact of microbiota at later life stages, gut microbiome homogenate transfers were carried out in recipient Canton-S female flies between 20 and 30 days of age. After the transfer, flies were kept on regular food for 5 days before total RNA extraction. Expression levels of both peptides were similar in flies that received young or middle-aged gut microbiome homogenates from *daGS>UAS Parkin* flies, regardless of induction status (Figures 4D,E). Interestingly, flies that received gut microbiome homogenates from old (day 60) uninduced Parkin donors showed significantly higher expression of Drosomycin and Diptericin compared to those that received gut microbiome homogenates from age-matched Parkin overexpressing flies (Figures 4D, E).

To further examine how the immune system of Canton-S flies responds to gut microbiome homogenates of different ages, we performed similar transfers using gut microbiome homogenates from young (day 10) and middle-aged (day 20) Canton-S donors. Flies that received the middle-aged gut microbiome homogenates showed significantly higher expression of both antimicrobial peptides compared to those that received the young gut microbiome homogenates (Figures 4A,B). These results indicate that the old microbiome activates the immune response in wild-type flies and that Parkin induction shifts the gut microbiota composition to prevent the age-related immune response activation.

## 4 Discussion

Our study provides the first evidence that overexpression of the *Parkin* gene (Parkin OE) alters gut microbiome composition in *D. melanogaster*, with implications for host lifespan and immune regulation. While prior research has explored microbiome alterations in Parkinson’s disease patients and animal models (Forero-Rodríguez et al., 2021; Gallop et al., 2021; Kang et al., 2021; Hill-Burns et al., 2017; Elfil et al., 2020; Parker-Character et al., 2021; Morais et al., 2024), this is the first study to assess microbiome dynamics in a Parkin OE model with improved longevity and healthspan. Our findings indicate that Parkin OE induces age-dependent microbial shifts and may contribute to healthier aging through modulation of gut microbial ecology.

Our results show that Parkin OE leads to distinct shifts in the gut microbiome compared to control flies. We observed differences in the composition of the gut microbiota across samples, as revealed by 16S rRNA sequencing data (Figure 2). These observations expand on previous studies showing that Parkin helps maintain mitochondrial function and increases lifespan in *Drosophila* (Si et al., 2019; Rana et al., 2013). We now show that Parkin OE is also associated with a less inflammatory gut microbiome during aging.

In the gut microbiome transfer experiments, germ-free flies that received microbiota from older Parkin uninduced flies showed reduced lifespan (Figure 3). By contrast, the lifespan of flies that received microbiota from Parkin induced donors remained stable regardless of donor age. These findings suggest that the Parkin-associated microbiome may preserve beneficial properties throughout the aging process. This effect was also reflected in the immune response. Expression of antimicrobial peptides such as Drosomycin and Diptericin increased in flies receiving older microbiota from control donors, but not in those receiving microbiota from Parkin OE donors (Figures 4D,E). These findings are consistent with studies showing that microbial changes during aging can lead to overactivation of immune pathways and tissue damage (Onuma et al., 2023; Hanson and Lemaitre, 2020). It is important to acknowledge, however, that it is possible that other components of the homogenate may contribute to the observed phenotypes.

To prepare germ-free flies for microbiome transfer, we chose antibiotic treatment rather than the conventional dechorionation method. This decision was based on practical and biological reasons. First, flies raised on antibiotics showed normal lifespans, similar to those raised on untreated food. Second, we found that dechorionation led to high mortality and reduced viability, making it difficult to generate enough individuals for survival and molecular assays (Koyle et al., 2016; Parker-Character et al., 2021). Re-association of the microbiome was done as previously described in (Clark et al., 2015). The rationale for adopting this method, where germ-free flies were fed with the whole microbiome from *Drosophila* at different stages, was to see whether the entire community could influence lifespan and healthspan. This approach also allowed us to include species that could not be cultivated in the lab, while screening, cultivating, and testing each bacterial species individually or in combinations would have been extremely time-consuming and not practical for this project. A similar strategy has also been used in more recent work, for example in the study ‘Feeding *Drosophila* gut microbiomes from young and old flies modifies the microbiome (Wesseltoft et al., 2024).

We found that both age and genetic background significantly influence gut microbial composition, with clear shifts observed across different life stages. These results align with earlier reports describing age-associated changes in microbial diversity and structure in *Drosophila* (Clark et al., 2015; Broderick and Lemaitre, 2012). Consistent with previous findings, we observed a decline in bacterial genera such as *Unclassified Gluconacetobacter, Gluconobacter, Serratia* and *Orbus* in older flies, while *Acetobacter pasteurianus, Acetobacter syzygii* and *L. brevis* became more prominent. *Lactobacillus brevis* was notably enriched in aged Parkin-overexpressing flies, suggesting that Parkin may support the expansion of beneficial microbes later in life. Interestingly, previous work found *L. brevis* to be more abundant in young flies under normal conditions (Lee et al., 2019). *Lactobacillus brevis* has demonstrated notable probiotic properties, including antimicrobial activity against various foodborne pathogens and strong antioxidant capacity in fermented skim milk, supporting its potential application as an adjunct culture in synbiotic-fermented dairy products (Kariyawasam et al., 2020). In addition, studies in aged mice have shown that *L. brevis* can help rebalance the gut microbiota by lowering the Firmicutes to Bacteroidetes and Proteobacteria to Bacteroidetes ratios, which tend to rise with age. It also reduced the levels of key senescence markers like p16, p53, and SAMHD1 in both the colon and the hippocampus, suggesting potential benefits for healthy aging (Jeong et al., 2016).

This pattern supports the idea that aging is associated with compositional restructuring of the gut microbiota, and that host genetics can influence these transitions to mitigate age-related dysbiosis. An increase in microbial load has been associated with aging and reduced lifespan (Lee et al., 2019). In older flies, this shift is typically characterized by a decrease in *Lactobacillus* species and an increase in *Acetobacter* abundance (Clark et al., 2015). However, this pattern is not observed in aged Parkin-overexpressing flies, where *Lactobacillus* remains a dominant genus. In addition, microbial diversity was better preserved in Parkin OE flies during aging. This preservation of microbial richness may reflect more stable host–microbiota interactions in Parkin OE flies, contributing to improved intestinal homeostasis and healthier aging trajectories. Further studies are needed to determine whether this increased diversity contributes to improved host outcomes or simply reflects microbial turnover.

The microbiome’s role in regulating host immunity was further supported by our antimicrobial peptide analysis. Older microbiomes from control flies triggered strong immune responses, consistent with age-associated activation of the Imd and Toll signaling pathways. These pathways respond to microbial cues and are known to become chronically activated with age, contributing to inflammation and functional decline (Onuma et al., 2023; Hearps et al., 2012; Ramsden et al., 2008). We found that Parkin OE microbiomes, even when taken from aged flies, did not trigger this immune activation. This suggests that Parkin OE helps maintain a microbial profile that is less likely to induce host inflammation. Overall, our findings support a model where Parkin contributes to host health not only through its known role in mitochondrial quality control but also by influencing gut microbiota composition and function. The Parkin-associated microbiome appears less inflammatory and more functionally stable with age. This dual effect may help explain the extended lifespan and preserved physiological function observed in Parkin OE flies. That said, we are aware that by feeding bulk homogenates, it is possible that additional components beyond the microbiome play a role in observed phenotypes. This means that the effects we observed could reflect not only the microbial communities themselves but also other components of the gut environment. In addition, we did not directly confirm how bacterial composition in recipient flies changed after feeding. While these factors do not affect the main conclusions of our work, future studies using defined bacterial consortia or direct microbiome profiling after transfer would help clarify the specific microbial contributions to the effects we observed on lifespan and immune activation.

While most other related studies have focused on the dysbiotic gut microbiome in Parkinson’s disease models (Li et al., 2019), altered microbiome composition has been observed in patients with PD, supporting the notion that a proinflammatory gut microbiome environment may act as a trigger for PD pathogenesis (Gorecki et al., 2019). In parallel, other studies have extensively investigated the effects of Parkin overexpression, such as its ability to attenuate ageing-related loss of muscle mass and strength and unexpectedly cause hypertrophy in adult skeletal muscles (Leduc-Gaudet et al., 2019). Additional reports have shown that Parkin overexpression reduces Drosophila Mitofusin levels in aging flies, with concomitant changes in mitochondrial morphology and increased mitochondrial activity (Rana et al., 2013). Parkin overexpression also reversed the accumulation of p62 in mitochondria, thereby restoring impaired mitophagy in Aβ-treated cells (Wang et al., 2020). Moreover, it exerted a significant protective effect on retinal ganglion cells (RGCs) and partially restored mitophagy dysfunction in response to cumulative intraocular pressure (IOP) elevation (Dai et al., 2018). However, to our knowledge, this is the first study to use Parkin overexpression to explore potential Parkin-related changes in the gut microbial community, changes that were captured dynamically at four different stages of the *D. melanogaster* lifespan.

These results open new directions for research on host–microbe interactions and aging, suggesting that targeting pathways involved in mitochondrial quality control may also benefit the gut microbiome and overall host health. They further provide a basis for exploring targeted interventions, such as probiotic administration, to help alleviate age-related effects.

## 5 Conclusion

This study shows for the first time that overexpressing the Parkin gene changes the gut microbiome in *D. melanogaster* as the flies age. We found that Parkin overexpression leads to age-related shifts in gut bacteria, helps preserve microbial diversity, and reduces immune activation, factors that likely support healthier aging. Flies with Parkin overexpression had a more stable and less inflammatory gut microbiome, and this beneficial effect was also seen when their microbiota was transferred to germ-free flies. These results suggest that Parkin supports host health not only by regulating mitochondria, but also by shaping the gut microbiome.

## Data Availability

The datasets presented in this study are publicly available. This data can be found here: https://www.ncbi.nlm.nih.gov/sra, accession number PRJNA1287252.
