# Supplementary material for: Parkin overexpression modulates gut-microbiota composition during aging in Drosophila melanogaster
Source: Front Microbiol. 2025 Sep 30;16:1672083. doi: 10.3389/fmicb.2025.1672083 (PMC12518341; doi:10.3389/fmicb.2025.1672083)
Supplement: Supplementary file 1 [file Data_Sheet_1.PDF]

Supplementary Figure 1. Bacterial abundance in *daGS>UAS-Parkin* control and Parkin-overexpressing flies.

qPCR analysis of (a) total 16S rRNA, (b) *Acetobacter*, (c) *Enterobacter*, and (d) *Lactobacillus* at days 10, 30, 45, and 60. Parkin overexpression was induced from day 1. Significant differences were observed for total 16S (day 60, p = 0.0045), *Acetobacter* (day 10, p = 0.0281; day 60, p = 0.0002), *Enterobacter* (day 10, p = 0.0095; day 30, p = 0.0026), and *Lactobacillus* (day 10, p = 0.0012; day 30, p = 0.0218). Data represent five biological replicates per condition, each comprising five female flies.

Supplementary Figure 2. Microbiome composition in *W1118/daGS* control and RU486-treated flies.

Bacterial abundance was measured by qPCR for (a) total 16S rRNA gene, (b) *Acetobacter*, (c) *Enterobacter*, and (d) *Lactobacillus*. Samples were collected from the *W1118/daGS* strain at four time points (days 10, 30, 45, and 60) under both control (untreated) and RU486-treated conditions. No significant differences were observed between control and RU486-treated groups of the same age. Panels A–D represent data from five biological replicates per condition, with each replicate consisting of five biologically independent *W1118/daGS* female flies.

Supplementary Table 1. Taxonomic summary of 16S rRNA data generated with Metaxa2. This table presents the taxonomic classification of bacterial sequences identified from 16S rRNA gene profiling across all samples and experimental conditions. Data include relative abundances at phylum, family, and genus levels for *daGS>UAS-Parkin* control and induced flies at four time points (days 10, 30, 45, and 60). The table summarizes taxa detected, overall bacterial composition, and variation across age and treatment conditions.
