# Supplementary figures and images for: Parkin overexpression modulates gut-microbiota composition during aging in Drosophila melanogaster
Source: Front Microbiol. 2025 Sep 30;16:1672083. doi: 10.3389/fmicb.2025.1672083 (PMC12518341; doi:10.3389/fmicb.2025.1672083)

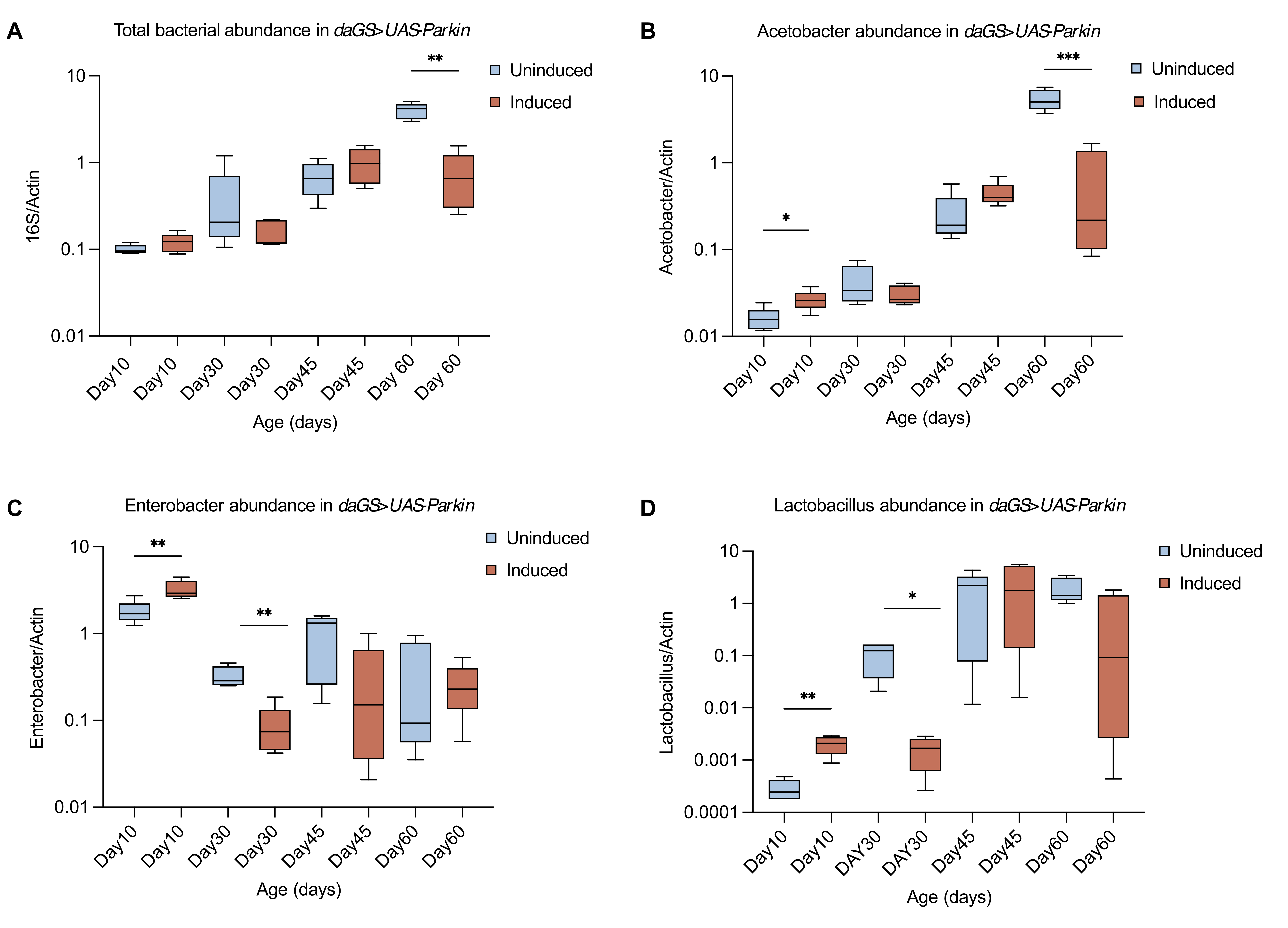

Supplement: Supplementary file 2 [file Image_1.TIFF]

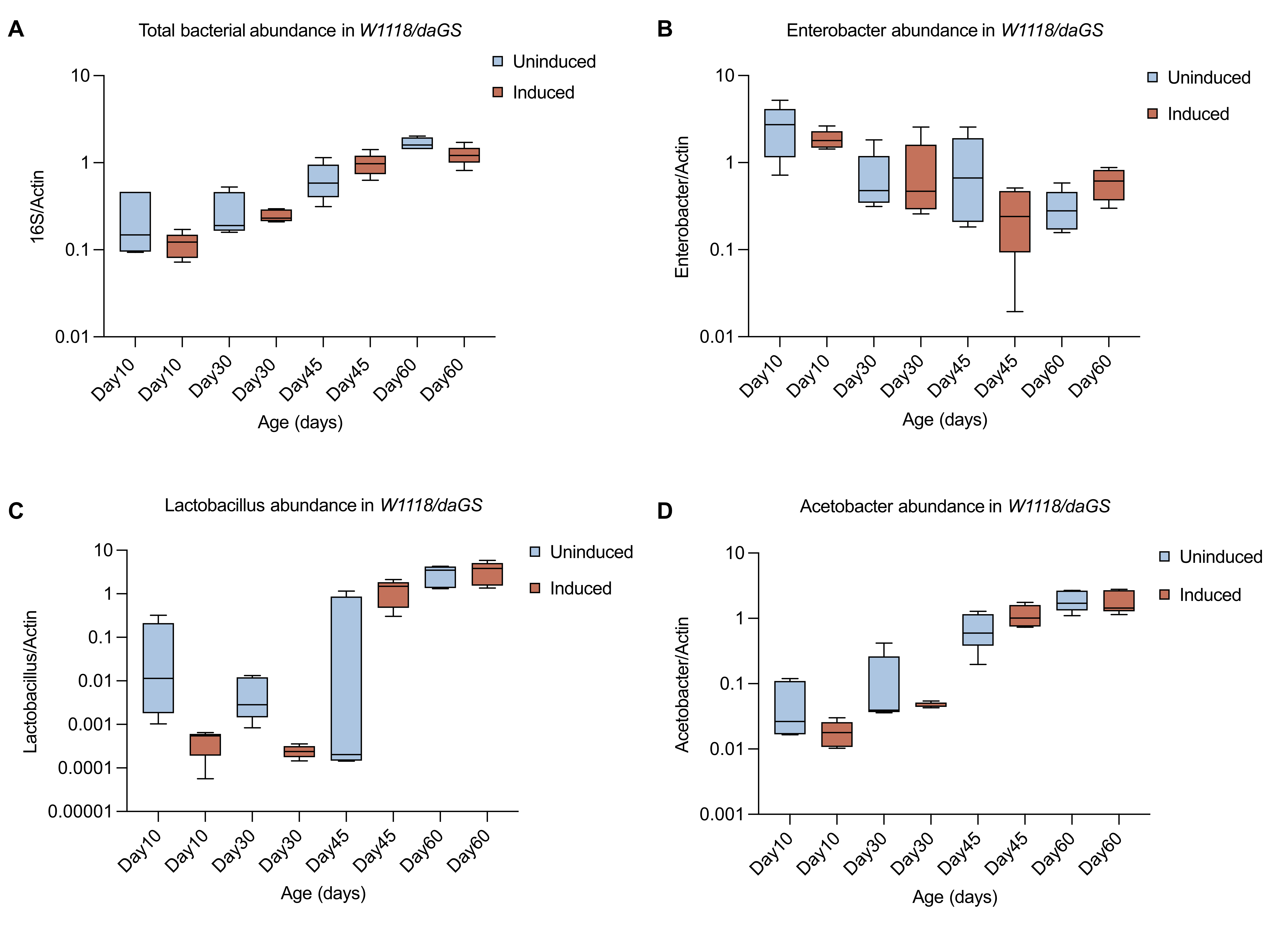

Supplement: Supplementary file 3 [file Image_2.TIFF]
